# Type Strains of Entomopathogenic Nematode-Symbiotic Bacterium Species, *Xenorhabdus szentirmaii* (EMC) and *X. budapestensis* (EMA), Are Exceptional Sources of Non-Ribosomal Templated, Large-Target-Spectral, Thermotolerant-Antimicrobial Peptides (by Both), and Iodinin (by EMC)

**DOI:** 10.3390/pathogens11030342

**Published:** 2022-03-11

**Authors:** András Fodor, Maxime Gualtieri, Matthias Zeller, Eustachio Tarasco, Michael G. Klein, Andrea M. Fodor, Leroy Haynes, Katalin Lengyel, Steven A. Forst, Ghazala M. Furgani, Levente Karaffa, Tibor Vellai

**Affiliations:** 1 Department of Genetics, Eötvös University, Pázmány Péter Sétány 1/C, H-1117 Budapest, Hungary; andrea.kaszas1@gmail.com (A.M.F.); katalinalengyel@gmail.com (K.L.); or g.alf@uot.edu.ly (G.M.F.); or vellai.tibor@ttk.elte.hu (T.V.); 2 Department of Genetics, University of Szeged, Középfasor 52, H-6726 Szeged, Hungary; 3 Nosopharm, 110 Allée Charles Babbage, Espace Innovation 2, 30000 Nîmes, France; m.gualtieri@nosopharm.com; 4 Department of Chemistry, Purdue University, 560 Oval Drive, West Lafayette, IN 47906, USA; zeller4@purdue.edu; 5 Department of Soil, Plant and Food Sciences, University of Bari “Aldo Moro”, Via Amendola 165/A, 70126 Bari, Italy; eustachio.tarasco@uniba.it; 6 Institute for Sustainable Plant Protection of CNR, Via Amendola 122/D, 70126 Bari, Italy; 7 USDA-ARS & Department of Entomology, The Ohio State University, 13416 Claremont Ave, Cleveland, OH 44130, USA; klein.10@osu.edu; 8 Department of Chemistry, The College of Wooster, Wooster, OH 44691, USA; haynes@wooster.edu; 9 National Institute of Pharmacy and Nutrition (NIPN), Zrinyi utca 3, H-1051 Budapest, Hungary; 10 Department of Biological Sciences, University of Wisconsin-Milwaukee, P.O. Box 413, Milwaukee, WI 53201, USA; sforst@uwm.edu; 11 Department of Plant Protection, Faculty of Agriculture, University of Tripoli, Tripoli P.O. Box 13793, Libya; 12 Department of Biochemical Engineering, Faculty of Science and Technology, University of Debrecen, Egyetem Tér 1, H-4032 Debrecen, Hungary; levente.karaffa@science.unideb.hu; 13 Institute of Metagenomics, University of Debrecen, Egyetem tér 1, H-4032 Debrecen, Hungary; 14 MTA-ELTE Genetics Research Group, Pázmány Péter Sétány 1/C, H-1117 Budapest, Hungary

**Keywords:** *Xenorhabdus* 1, NRP-AMP 2, fabclavine 3, iodinin 4, exocrystal 5, phenazine 6, PAX-peptides 7, szentiamide 8, R-type bacteriocins 9, EPN/EPB cospeciation 10

## Abstract

Antimicrobial multidrug resistance (MDR) is a global challenge, not only for public health, but also for sustainable agriculture. Antibiotics used in humans should be ruled out for use in veterinary or agricultural settings. Applying antimicrobial peptide (AMP) molecules, produced by soil-born organisms for protecting (soil-born) plants, seems a preferable alternative. The natural role of peptide-antimicrobials, produced by the prokaryotic partner of entomopathogenic-nematode/bacterium (EPN/EPB) symbiotic associations, is to sustain monoxenic conditions for the EPB in the gut of the semi-anabiotic infective dauer juvenile (IJ) EPN. They keep pathobiome conditions balanced for the EPN/EPB complex in polyxenic (soil, vanquished insect cadaver) niches. *Xenorhabdus szentirmaii* DSM16338(T) (EMC), and *X. budapestensis* DSM16342(T) (EMA), are the respective natural symbionts of EPN species *Steinernema rarum* and S. *bicornutum.* We identified and characterized both of these 15 years ago. The functional annotation of the draft genome of EMC revealed 71 genes encoding non-ribosomal peptide synthases, and polyketide synthases. The large spatial *Xenorhabdus* AMP (fabclavine), was discovered in EMA, and its biosynthetic pathway in EMC. The AMPs produced by EMA and EMC are promising candidates for controlling MDR prokaryotic and eukaryotic pathogens (bacteria, oomycetes, fungi, protozoa). EMC releases large quantity of iodinin (1,6-dihydroxyphenazine 5,10-dioxide) in a water-soluble form into the media, where it condenses to form spectacular water-insoluble, macroscopic crystals. This review evaluates the scientific impact of international research on EMA and EMC.

## 1. Introduction

Antimicrobial multidrug-resistance (MDR) [1] is an indirect consequence of large-scale and non-professional applications of previously powerful antibiotics, leading to the situation in which the lifesaving role of antibiotics has gradually become diminished [2]. MDR has become not only a global public health concern, but also a challenge for sustainable agriculture [3] and plant health management problems [4]. As for plant and veterinary health aspects, the situation is exacerbated by the fact that those antibiotics which are of use (or of potential use) in human clinical practice should unambiguously be ruled out in a veterinary or agro-business setting [5].

Since wild type as well as most cultured plants are by definition “soil-born” organisms, the concept of battling MDR plant pathogens with natural antimicrobial peptides (AMPs) produced by other soil-born organisms was taken into consideration. The soil-borne entomopathogenic bacterium (EPB), a symbiont of soil-born insect pathogenic nematodes (EPN) [6], is an AMP-producing organism. For the definition of AMP, we consider peptides as any polyamide, or even biopolymer, with an ester, thioester, or otherwise modified backbone, that can be made on a contemporary chemical peptide synthesizer [7]. In this Review, we do not deal with *Xenorhabdus*-produced antibiotics other than AMP. The AMP products are to protect their eukaryote (also “soil-born”) symbiotic partners from both prokaryotic and eukaryotic pathogenic competitors present around them in their respective niche. EPB-released AMP products provide a monoxenic milieu in the gut of the EPN infective juvenile (IJ) for the non-propagating EPB symbiont [6]. This keeps the pathobiome conditions optimally balanced [8] for the EPN/EPB symbiotic complex in the polyxenic colonized insect cadaver and soil. Therefore, they are potential sources of compounds with MDR-control capabilities. Some special AMP molecules in symbiotic associations can act as regulatory molecules, or serve in communication between the symbiotic partners, but these functions are outside of the scope of this review.

Literature from the last 15 years indicates a trend within EPN/EPB research in this antimicrobial direction. This conception led us 15 years ago to search for, and finally find, isolate, identify, characterize, and deposit, two beneficial AMP-producing EPB strains [9] under the respective names *Xenorhabdus szentirmaii* nov. DSM16338(T), (lab strain-code EMC) from the South-American *Steinernema rarum* [10] EPN species; and *X. budapestensis* nov. DSM16342(T), (lab strain-code EMA), from the Central European *S.* *bicornutum* [11] EPB species [9]. Unless otherwise noted, all *X. szentirmaii* and *X. budapestensis* mentioned in this review are the type strains noted in [9]. All EMA and EMC were isolated from the EPN collection in our laboratory at the Department of Genetics, Eötvös University, Budapest, Hungary [12] Stackabrrandt et al., 2021.

Another two EPB species were also identified and published in a previous article [9]. *Xenorhabdus innexii* DSM16336(T) [9], from the cricket pathogen *S. scapteriscii* [13], later proved to be a strong insecticide producer [14]. *Xenorhabdus innexii* DSM16336(T) may be conspecific with *Xenorhabdus* strain UY61 [15], and is known to establish an experimentally reproducible, cricket-specific, lethal combination, but is not very pathogenic against Lepidoptera [15]. The fourth EPN discovered was *X. ehlersi* DSM16337(T) [9] from *S. serratum*, (Byron Adams, personal communication), but it is also known as a natural symbiont of *S. longicaudatum*, representing EPB species which are capable of inactivating the cellular immune mechanisms of the attacked insect [16,17].

This review aims to summarize the research history and scientific impact of the research efforts on our isolates. *Xenorhabdus szentirmaii* sp. nov., *type strain* DSM 16338T, and *Xenorhabdus budapestensis* sp. nov., DSM 16342T, may be scored as among the best AMP producing EPB species.

Our “didactic” approach is an attempt to guide the Reader through the “chapters and subchapters” of an imagined “virtual book” about EPN-EPB research. We believe that the results obtained from experiments on EMA and EMC globally have significantly contributed to this research. Most, but not all, of these results came from labs other than our own.

The latest high-impact publications on EMA and EMC came from the Bode Laboratory (Frankfurt, Germany). We refer to and cite our pioneering works [9], and to detailed personal communications via the COST 819 and COST 850 European Joint Research Actions. Sebastian Fuchs and his associates were able to isolate the most efficient antimicrobial active AMP compound from the cell-free conditioned medium (CFCM) of our type strains noted above [18], excluded the previously suggested bicornutin oligopeptide as a possible AMP compound (based on A. Patthy personal communication) by Böszörményi et al. [19], and instead identified it as fabclavine [20]. Fabclavine was later shown to be the key AMP product of the most efficient antimicrobial-producing *Xenorhabdus* species [21,22]. In fact, an analog of the peptidic part of each fabclavine was discovered in *X. cabanillasii* prior to later findings, and was patented under the name nemaucin [23]. Similarly, referring to our own work [24], and based on detailed personal communications with American fellow scientists, Brachmann and his associates revealed the phenazine biosynthesis pathway [25] in the only iodinin-producing *Xenorhabdus* strain [24], *X*. *szentirmaii*.

## 2. Agricultural Aspects of Multidrug Resistance (MDR)

### 2.1. Antimicrobial Peptides as Tools to Beat MDR Pathogens

The usage of the new arsenal of peptide antibiotics in the battle against MDR pathogens is of emerging therapeutic potential [26], since many newly appearing MDR organisms seem to show collateral sensitivity [27,28]. Furthermore, AMP resistance and antibiotic resistance genes differ in their mobilization patterns and functional compatibilities with new bacterial hosts [29]. The various AMP molecules differ considerably concerning their physicochemical properties and cellular targets, as well as their resistance determinants [30]. Cross-resistance between AMPs appears to be rather rare [31]. Furthermore, the co-evolutionary trends of resistance against antimicrobial peptides [32], and those against conventional antibiotics, must also be different [31].

### 2.2. Changes in the Scope of the EPN/EPB Research Due to the Perspectives of EPB-Produced AMPs in Combatting MDR Pathogens

Similar to the research trends on entomopathogenic fungi [33,34], those related to EPN/EPB symbiotic associations have been restricted to biological insect pest control tools for sustainable agriculture [35,36,37,38,39,40,41,42,43,44,45,46,47].

The antibiotic-related perspectives were recognized [48] only when the global threat of MDR became obvious [49], although the antibiotic-productive capabilities of the obligate Gram-negative bacterial symbionts belonging to the *Xenorhabdus* and *Photorhabdus* genera of EPN strains, or belonging to species of the *Steinernema* and *Heterorhabditis* genera, had been known since 1972 [50,51,52,53,54].

Indispensable subchapters of the history of EPN/EPB research are those which revealed the detailed mechanisms and coevolutionary aspects of the symbioses [55,56], the unique unprecedented epigenetic mechanism called the primary/secondary (mostly) irreversible phenotypic phase shift both in *Xenorhabdus* and *Photorhabdus* [54,55,56,57,58], and the coevolutionary aspects [59,60,61,62,63,64,65,66,67,68,69], including our own contributions.

### 2.3. How Do Antibiotic-Producing EPN/EPB Symbioses Work?

**Figure 1:**
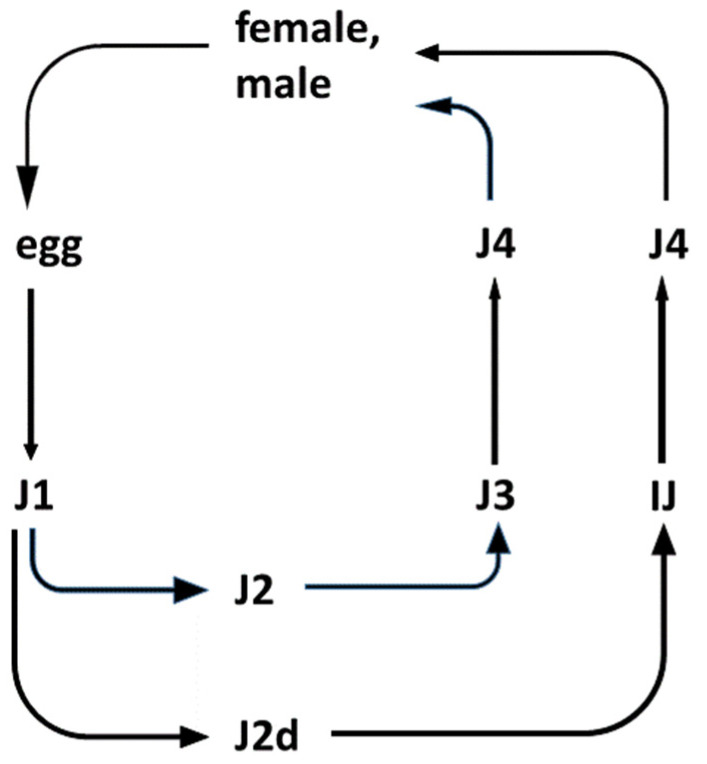
Life cycles of entomopathogenic *Steinernema* nematode species. (The life-cycle of *Heterorhabditis* is not shown). **Legend**: The outlines of the life-cycle of EPN species belonging to the *Steinernema* genus. There are six postembryonic developmental (juvenile, larval) stages (J1, J2, J2d, J3, J4, and IJ: J = juvenile; I = infective; and IJ = infective dauer, or the enduring, non-aging, non-feeding, non-growing, semi-anabiotic larva [72]. In nature, only the IJ can be found in the soil outside the insect cadaver. They are capable of entering the insects through their natural openings, and infecting them. Immediately after entry into the insect, the pharynx of the IJ starts to pump, releasing their symbiotic bacteria through their mouths into the hemocoel of the insect. The bacteria propagate rapidly and release toxins, killing the insect host. Meanwhile, the IJ molts, and develops into a J4. Adults develop from the J4 in the insect cadaver. In the case of *Steinernema*, the adults are 50/50 female/male. Males then fertilize the females. In the case of *Heterorhabditis*, self-fertilizing adult hermaphrodites develop from the infecting IJ. Most of their eggs develop inside the hermaphrodite (called “endotoxin matricida”), and the majority develop into females and males. Only a small fraction grows to additional self-fertilizing hermaphrodites. After 2–3 cycles, the concentration of a secondary metabolite of a lipid nature, which serves as a genus-specific chemical developmental signal, reaches a level that induces an altered developmental pathway for the J1 larvae to develop to an IJ through a special second (J2d) developmental stage. IJs leave the cadaver and search for new insect hosts, aided by chemo-attraction.

Insects, EPN, and EPB are capable of forming a tripartite relationship called mutualism [70]. This includes a host/parasite relationship between the EPN and the infested insect prey; a host/pathogen relationship between the colonized insect prey and the EPB pathogen; and finally a symbiotic relationship between the respective EPN and EPB [71], as demonstrated in Figure 1.

The EPN/EPB symbiosis is taxon-specific. Whereas the *Steinernema* EPNs can only establish symbiosis with bacteria belonging to the genus *Xenorhabdus*, EPNs in genus *Heterorhabditis* can only establish symbiosis with *Photorhabdus* bacteria. The dauer juvenile (IJ) nematodes store, with few known exceptions, the respective symbiont monoxenically in their guts. The feeding forms (J1, J2, J2d, J3, J4, and adults) consume the bacteria together with the bacterium-digested insect tissues.

### 2.4. The Natural Role of the EPB

**Figure 2:**
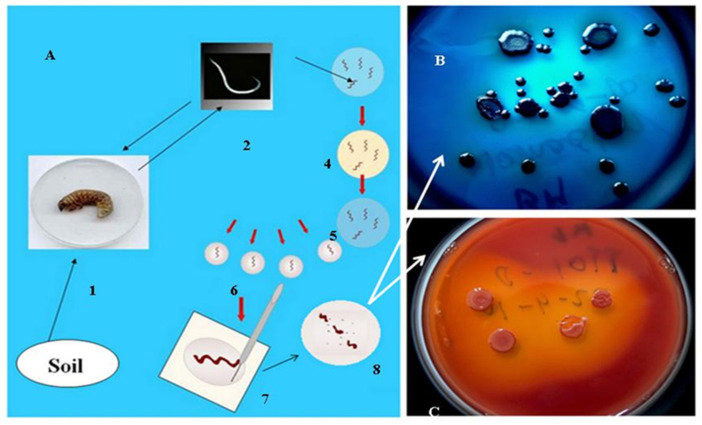
Isolation of EPB cells from IJs (**A**) by Lucskai’s bleach method. Infective dauer juveniles, either from the soil, or insect cadaver, are surface sterilized in HOCL, put in a sterile physiological salt solution in a sterile Petri dish, cut into pieces, and μL volumes from the saline solution are dropped in agar (LBA) media, (**C**) seeded, and incubated at 25 °C for one day. Fresh colonies are picked and transferred to an indicator (LBTA) plate (**B**) where the Phase I DPN cells can be unambiguously recognized and cloned. (For details, see Appendix A).

As mentioned in the introduction, the biological role of antimicrobial, mainly peptide, products of the EPB is to provide, establish and sustain monoxenic or balanced pathobiome conditions for the respective nematode/bacterium symbiotic complex in a polyxenic environment, such as the cadaver of the vanquished insect in the soil, in the niche where they live [8,56,57]. More accurately, during our long-term observations, whenever the EPB was isolated in sterile conditions from the gut of surface sterilized IJs [9], we found only one single bacterium species, and this was the respective EPB symbiont in the primary (phase I, 1) form, in agreement with [56,57], see Figure 2.

However, whenever we tried to isolate EPB from an infected insect cadaver, we never found them monoxenically, in agreement with [8].

**Figure 3:**
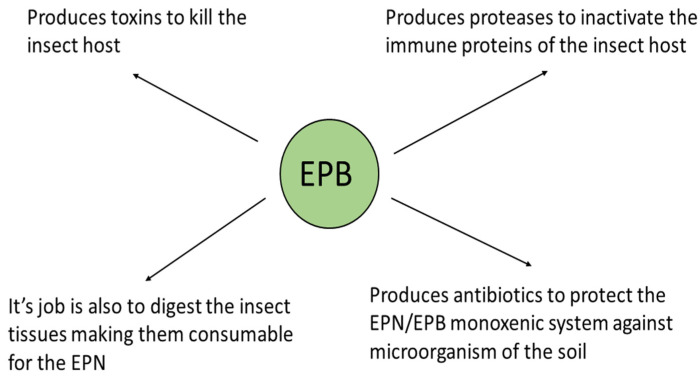
The biological role of the EPB symbiont in nature. (Presented by Fodor et al. BABE-2015 6th World Congress on Bioavailability & Bioequivalence: BA/BE Studies Summit 17–19 August 2015).

The triple role of the EPB symbiont is as follows: (1) producing insect-killing toxins [73,74,75], (“serving like a soldier”); (2) digesting the insect tissues making them consumable for the EPN, (“acting like a cook”); and (3) as a producer of antimicrobial peptides to protect the EPN/EPB symbiotic complex from competitors existing in the polyxenic soil, (“serving like a bodyguard”, [50,51,52,53]). This is schematically summarized in Figure 3.

Immediately after entering the insect body cavity through the natural openings of the insect, the IJ releases the EPB into the hemocoel, where it starts to propagate and produce toxins of a protein nature, causing lethal septicemia in the insect prey, and decomposing the insect tissue. This tissue becomes consumable for the propagating EPN population, and releases antimicrobials to provide balanced, probiotic, conditions for the symbiotic complex, the polyxenic cadaver, and the soil [8].

## 3. Coevolution and Co-Speciation of EPN/EPB Symbiotic Associations

Except for the human pathogenic *Photorhabdus asymbiotica* [76,77,78], no EPB bacteria can be found in the soil as a free-living organism, but only in the colonized insect cadavers, and the monoxenically colonized gut of the infective dauer juvenile (IJ) developmental variant EPB [56], as symbiotic partners of the respective EPN. Many EPN/EPB associations have been discovered so far. Two EPN genera (*Steinernema*, *Heterorhabditis*), and 2 EPB genera (*Xenorhabdus*, *Photorhabdus*) are involved. Each EPN and EPB genus includes several species, subspecies, and strains.

Each *Steinernema* EPN strain is capable of establishing symbiosis with one or more, but a very limited number of, *Xenorhabdus* strains, and exclusively with *Xenorhabdus*, with no exception [57], which usually, but not exclusively, belong to the same species or subspecies.

**Figure 4:**
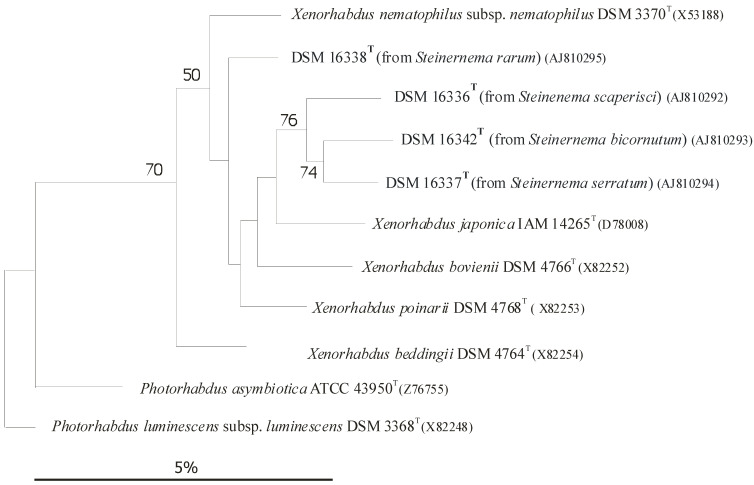
Dendrogram of 16S rRNA gene sequence similarities of *Xenorhabdus* species generated by distance matrix analysis. **References**: [60] References to Figure 4: [61,62,63]. **Caption:** (After [9] Figure 1): Dendrogram of 16S rRNA gene sequence similarities generated by distance matrix analysis. Figure 4 demonstrates the close taxonomic relation between *Xenorhabdus* and *Photorhabdus* genera. The taxonomic joining point of the two genera was also discovered by us as a part of the reviewed project; see [59,60]. The sequence of *Proteus vulgaris* served as the root. Bar = 2 nucleotide substitutions per 100 nucleotides. The numbers are bootstrap values. Similarly, we constructed a dendrogram for the genus *Photorhabdus* (not shown) and defined Subclusters [59,60], which were later reconstructed more accurately, and obtained a subspecies rank [66,68].

Each *Heterorhabditis* EPN strain is capable of establishing symbiosis with one or more, but a very limited, number of *Photorhabdus* strains, but exclusively with *Photorhabdus*, with no exception [56]), usually, but not exclusively, belonging to the same species or subspecies. In the case of EPB species, the rDNA sequence-based subclusters [59,60], more-or-less correspond to subspecies rank [66,68]), as demonstrated in (Figure 4) [61,62,63,64,65,66,67,68,69].

### 3.1. Gnotobiological Analysis as a Reliable Experimental Approach to Co-Speciation

**Figure A1:**
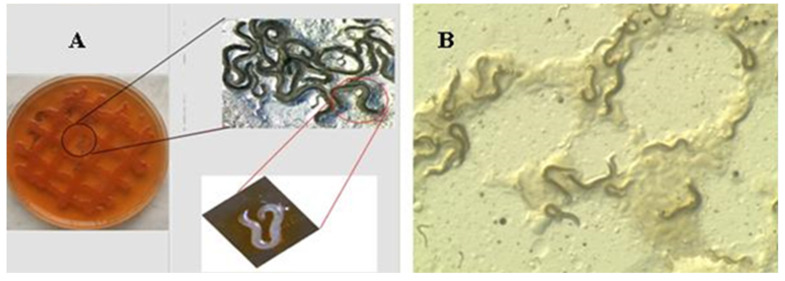
Entomopathogenic Nematode Growth (ENGM) Plate see ref. [86] (Photo: Csaba Pintér (**A**); Andrea Máthé-Fodor (**B**). Author> András Fodor.

The experimental approach to tracing trends in co-speciation [79] as a way of coevolution is gnotobiological analysis (a term from Professor N. E. Boemare, personal communication) carried out via international cooperation [15,67,80]. The reliable gnotobiological analysis is based on experimental exchanges of molecular taxonomically identified EPB symbionts between molecular taxonomically identified EPN strains [15]. Apart from the sequence and the polyphasic taxonomy-based unambiguous identifications of both the prokaryote and the eukaryote symbiotic partners, there are three other essential preconditions needed for a reliable experimental gnotobiological analysis. (A) Isolation and establishing of a sterile monoxenic lab culture of the symbiotic EPB bacterium from its EPN partner. The only reliable source is the gut of surface-sterilized IJ of one of the EPN partners. We recommend the bleach technique established in our laboratory [9], and later also used by others [59,60,64,65], see Figure 2B Axenized eggs or IJs [81,82,83,84], from an EPN. (C) The availability of a special agar media for monoxenic culturing EPN on one’s own, or new, EPB symbiont, in transparent, visible, solid, media (similar to NGM, “Nematode Growth Media”, used for culturing *Caenorhabditis elegans*) by the *C. elegans* research community since Sydney Brenner’s report [85]. We can recommend our ENGM media for this application (see Appendix B, Figure A1). The ENGM is seeded by an EPB from the first EPN, and inoculated with an axenic J1 or IJ larvae from the second EPN [86] This is a reproducible method for symbiotic partner exchange studies, (see Appendix A and Appendix B).

### 3.2. Coevolution via Co-Speciation: Antimicrobial Active Peptides as Strategic Weapons Used in the Struggle to Conquest a Given Niche

In a given niche there are usually more than one EPN/EPB symbiotic complexes present and competing with each other if their insect targets are the same. The coevolution of interacting species can lead to codependent mutualists [71]. The precondition for evolutionary fixation of an EPN/EPB symbiotic complex in a given niche of a respective EPN/EPB complex is to win the struggle of insect prey against natural enemies, as well as competitors. Meanwhile, the mutualism should be kept [71]. Each symbiotic EPB (*Xenorhabdus*, *Photorhabdus*) partner owns an individual set of chemical arsenals for these unavoidable battles.

#### 3.2.1. Battle with the Insect Prey Using Toxins

A successful symbiotic complex needs to be able to kill the available insect prey more efficiently than other alternatives. For this, the EPB should produce toxins [87,88,89,90,91].

#### 3.2.2. Battle with EPN Competitors Using Rhabdopeptides

Seven linear peptides named rhabdopeptides I-O, 1–7, were recently isolated from the cell-free culture media (CFCM) of *X. budapestensis* SN84 [92]. The structures of the peptides were elucidated based on extensive mass spectrometry (MS), and nuclear magnetic resonance (NMR), analyses. Rhabdopeptides I-3, rhabdopeptides I-4, and rhabdo-peptides I-7 were novel compounds. All seven compounds were tested for their nematicidal activities against the second-stage juveniles (J2) of *Meloidogyne incognita*. Rhabdopeptide I-2 demonstrated strong inhibitory activity [92].

#### 3.2.3. Battle between Competitor EPBs Using Xenorhabdicins

Different *Steinernema* EPN species coexist with different *Xenorhabdus* symbionts when invading the same insect, setting up a competition for nutrients within the insect cadaver. The different *Xenorhabdus* species produce both diverse antibiotic compounds and prophage-derived R-type bacteriocins, xenorhabdicins [93]. The functions of these molecules during competition also seems extremely important from the aspect of coevolution.

**Figure 5:**
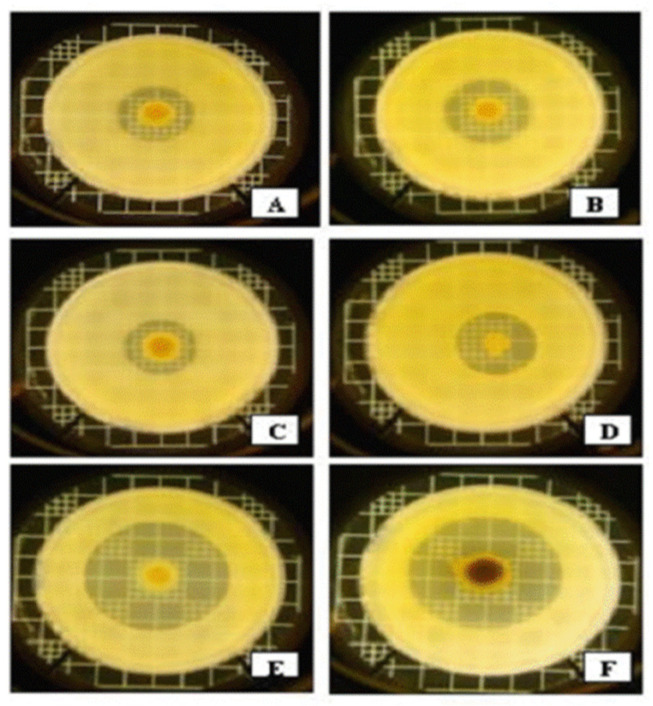
Interspecific differences in anti-Gram-negative activities within the genus *Xenorhabdus* based on overlay bioassays in LBA media on *Klebsiella pneumoniae* (mastitis isolates from cows). Legend to Figure 5: Each bacterium colony was grown from a 5 μL dropping of an overnight liquid (LB) culture on the surface of LBA medium for 5 days at 25 °C, and overlaid with 3 mL of soft (0.05 *w*/*v*) agar containing 0.3 mL log-phase (OD = 0.25) liquid (LB) culture of mastitis isolate *Kl. pneumoniae* in the Hogan laboratory at the Ohio State University, Wooster, OH, USA [96]. **A** = *X. nematophila* DSM3370; **B** = *X. cabanillasii* BP; **C** = *X. nematophila* ATCC 196061(T); **D** = *X. bovienii* NYH; **E** = *X. budapestensis* DSM16342(T); **F** = *X. szentirmaii* DSM16338(T), and cultured at 37 °C overnight. Note that by far the largest inactivation zones can be seen around the EMA colony (5E) and EMC (5F). Note the color of the EMC colony caused by iodinin crystals on the surface; see later).

Anti-*Xenorhabdus* activities of strains representing the 7 *Xenorhabdus* species against each other, and non-related Gram-negative bacteria, were compared in LB media [94]. The strongest anti-*Xenorhabdus* activity was shown by the CFCM of. *X. bovienii* NYH, (a symbiont of *S. feltiae*, isolated by AF in Nyíregyháza, Hungary) [95]. This showed a moderate antibacterial activity against Gram-negative bacteria *Escherichia coli* and *Klebsiella pneumoniae* (see Figure 5D), compared to other *Xenorhabdus* species.

The CFCM of *X. ehlersii* was also toxic to many other *Xenorhabdus*, but completely ineffective against *E. coli* OP50, or *Kl. pneumoniae*. On the other hand, the strongest antibiotic producers, *X. budapestensis* and *X. szentirmaii* (Figure 5E,F, respectively), were rather vulnerable to the anti-Xenorhabdus compounds produced by the others. Meanwhile, their compounds were barely effective against other *Xenorhabdus* species, at least on complete (LBA) media. *Xenorhabdus innexi*, a moderate anti-Gram-negative antibiotic producer, proved highly tolerant to the anti-*Xenorhabdus* compounds of others, with the exception of *X. bovienii* NYH [95].

The conclusion is that there was no correlation between the general anti-Gram negative and the anti-*Xenorhabdus* activities, but there was a positive correlation demonstrated between the anti-*Xenorhabdus* activities and sensitivity to anti-*Xenorhabdus* compounds in the CFCM [94].

10 years later in another experiment [97], using another *X. bovienii* strain, the natural symbiont of *S. jollieti*, (called Xb-Sj) was a very weak antibiotic producer. It possesses a P2-like phage tail gene cluster (xbp1), that encodes genes for xenorhabdicin production (Steven A. Forst, personal communication). Purified xenorhabdicins from the CFCM of *X. bovienii* Xb-Sj strain exerted a sharp, but narrow, spectrum of activity only towards *Xenorhabdus* and *Photorhabdus* species [97] (Thappeta et al., 2020).

In that experiment, *X. szentirmaii* was extremely sensitive towards the purified *X. bovienii* xenorhabdicin, and it did not produce effective xenorhabdicin against the *X. bovienii* Xb-Sj strain, at least not in poor Grace’s medium [97]. However, it was demonstrated that *X. szentirmaii* produced high-level antibiotic activity, which killed *X. bovienii* in a complete rich medium [97]. When the two species were co-cultured in either of the two media, *X. szentirmaii* was the winner. One can conclude that in nature the production of antibiotics is probably predominant in interspecies competition [98].

In the battle to win over food competitors by using AMPs and other secondary metabolites, the most successful symbiotic EPN/EPB complexes should be able to produce the best antimicrobial peptides to win against food-competitor microorganisms. This Review focuses on two molecule families.

### 3.3. Antimicrobial Peptides from EMC and EMA, Fabclavines from both, and Phenazines from EMC

There have been a few biosynthetic AMP families discovered in the *Xenorhabdus* species over the last decade, and providing the complete inventory of them is out of the scope of this review. Enzymes called ’non-ribosomal templalted peptide synthetizers’ (NRPSs) produce a wide variety of different natural peptid products from amino acid precursors [98]. These non-ribosomal encoded peptides (NRPs) are of short chain lengths. The common features of these molecular families are as follows. Each of them is a hybrid molecule, enzymatically synthesized by enzymes encoded by the members of a respective biosynthetic gene cluster (BGC) consisting of cooperating genes. The corresponding biosynthetic gene clusters (BGCs) could easily be identified by gene-sequence-similarity-based bioinformatics strategies [99]. Until recently, the actual access to these biosynthetic natural products for structure elucidation and bioactivity testing had been extremely difficult. The Bode laboratory recently discovered that the global post-transcriptional regulator, Hfq, which is widespread in bacteria and performs many functions, one of which is the facilitation of sRNA binding to target mRNAs, exerts several other pleiotropic effects [100]. A complete hfq deletion mutant EPB is no longer capable of sustaining a healthy symbiosis with its EPN partner due to the abolition of the production of all known secondary metabolites [100], i.e., the deletion of the gene encoding the RNA chaperone, Hfq, results in strains losing the production of most synthetic natural products, including NRPs [101]. Each contained a non-ribosomal-templated poly-amine (NRP) moiety. Each BGC encodes for one branch of nonribosomal peptide synthetases (NRPSs) [98,102,103]. In general, the NRPS consist of polypeptides, with a unidirectional interaction order, from N-terminal to C-terminal. There are usually adenylation domains, thiolation domains, condensation domains, dual condensation/epimerization domains, and thioesterase domains, involved (see [98], Supplementary Figure).

#### 3.3.1. The Most Potent NRP-AMP Families of Xenorhabdus Origin

**The Lysine-Rich, Cyclo-Lipopeptide, Molecular family**

This family was discovered in *X. nematophila* by a member of our team, M. Gualtieri, and his associates [99]. It is also called Peptide Antimicrobial and is of the *Xenorhabdus* species (PAX peptides is the name introduced by Thaler and the other members of that research team). The biosynthesis pathway of lysine-rich cyclic peptides in *X. nematophila* was made by the Bode team in Frankfurt, Germany [104].

**The fabclavine molecular family**

This extremely important molecular family was discovered in EMA (the type-strain of *X. budapestensis*), and iyd its biosynthesis pathway was discovered in EMC (the type-strain of *X. szentirmaii*). Fabclavine [18] was identified as a bioactive, non-ribosomal encoded (NRP) peptide-polyketide-polyamine hybrid [20]. As revealed by detailed NMR and MS methods, the fabclavine analogs are hybrid secondary metabolites derived from nonribosomal peptide synthetases (NRPS) and polyunsaturated fatty acids (PUFA) [105], [20]. As mentioned earlier, a structural analog, nemaucin [23], of the peptidic part of fabclavine was discovered by the Gualtieri team earlier from *X. cabanillasii* (Patent. WO2012085177A1, Nosopharm, Nîmes, France, 2012). It was published as an antibiotic compound purified from *X. cabanillasii* strain CNCM I-4418 [23].

Fabclavine derivatives could also be found in almost all known *Xenorhabdus* species, but the details of the enzymatic biosynthesis of fabclavine were revealed in *X. szentirmaii* by [21]. They used deletion mutants of the gene encoding the RNA *chaperone*, Hfq, and then by exchanging the native promoter of the fabclavine (fcl) BGC against an inducible promoter in Δhfq mutants, (easy PACId approach, easy Promoter Activated Compound Identification technique) [101], resulting in the exclusive production of the corresponding fabclavine from the targeted BGC in *X. szentirmaii* [21], and later in other *Xenorhabdus* species [22]. Altogether, 32 members of the fabclavine family are now known [22].

The fabclavine biosynthesis in different *Xenorhabdus* species is catalyzed by a very similar biosynthetic enzyme complex (Peptide-Antimicrobial *Xenorhabdus* Protein Synthetase) coded by biosynthesis gene clusters (BGC), including enzymes needed for polyamine synthesis [22]. Most *Xenorhabdus* species are capable of synthesizing fabclavine analogs in a rather conservative manner, and the genetic differences in amino acid sequences of the NRPS-PKS genes cannot explain the species-differences in antimicrobial activities.

It was suggested that differential virulence of *Xenorhabdus* strains (demonstrated in Figure 5) must be caused by the difference in the global leucine-responsive regulatory protein expression level metabolites [106,107,108,109,110], leading to a difference in the production of indole compounds, and other NRPS-PKS-associated secondary metabolites [106].

The antimicrobial peptides which are effective against intruder competitors (belonging to different prokaryotic and eukaryotic taxa) competing for the same environmental niche, serve as a powerful toolkit for promoting local co-evolutionarily fixation [111] of the respective EPN/EPB symbiotic complex.

#### 3.3.2. Iodinin and Phenazines

*Xenorhabdus szentirmaii* has extremely unusual phenotypes. One of them is their swarming behavior, and the other is exocrystal production [24]. Their motilities, both swimming and swarming, are much stronger than in any study published for a *Photorhabdus* or *Xenorhabdus* species [112,113,114,115,116,117].

**The Exo-Crystal of EMC, and the Iodinin Biosynthesis as a Part of the Phenazine Pathway.**

##### Basic Observation

**Figure 6:**
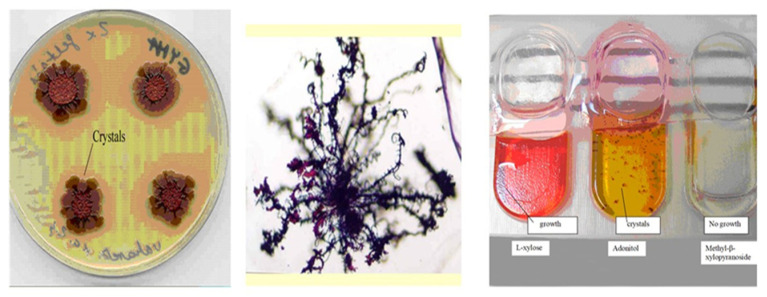
Formation of iodinin exocrystals on and under colonies of antibiotic producing *Xenorhabdus szentirmaii* DSM16338(T) (EMC). Crystals on agar plates (**left**) and in liquid cultures in (API) test tubes (**right**). (**Center**), 40× magnification (Jenaval Light Microscope).

Antibiotic pigment crystals were discovered and isolated by Máthé-Fodor in 2003, unpublished, but presented by Fodor et al. (BABE-2015 6th World Congress on Bioavailability & Bioequivalence: BA/BE Studies Summit 17–19 August 2015). An interesting phenomenon was discovered in the lab. After a few days of culturing *X. szentirmaii* on either NA, LBA, NBTA, or LBTA agar plates, the surfaces of the colonies became brilliant metallic red. At the same time, small crystals, as well as red colored oily drops, could be seen, first with a transmission light microscope, and later with the naked eye, both in the agar and liquid media (Figure 6).

The number and size of the crystals increases day by day. Crystals closer to the colonies were larger and continuous, whereas those located farther away were smaller and dendritic in nature (Figure 6, Center). On other media (ENGM, see Appendix B), large red-colored oily drops formed at the edges of the colonies. In solid media, the number of oily drops was higher closer to the center of the colonies, and lower farther out [24,86]. It appears that cells of *X. szentirmaii* release a precursor material that is water-soluble and colorless. When OUTSIDE of the cells, this material changes color and becomes water-insoluble, and separates, either dissolved in oil droplets, or crystallizing on the surface and inside the agar media. This red colored material was later found to be iodinin (5,10-dioxidophenazine-5,10-diium-1,6-diol) [118]. Iodinin is a well-known, natural, phenazine dioxide, compound that was recently “rediscovered” as, among others, possessing potent and selective cytotoxic properties towards myeloid leukemia cell lines [119,120,121,122], but the water-insolubility complicates clinical application [123], see Appendix C.

The colored oil droplets or pigment crystals form inside the agar medium, even if sterile cellophane separates the surface of the bacteria colonies from the agar. The cellophane Millipore 0.22 µm filter prevents the bacteria from passing into the agar, but iodinin still separates in the agar under those conditions. Two possible interpretations of this observation can be imagined. One of our team (L. Haynes) proposes that a water-soluble form of iodinin, rather than a chemically distinct precursor, could have been released by the cells. He proposes that the iodinin might be complexed by a water-soluble carbohydrate, which makes the complex water-soluble and gives it the ability to pass through the cellophane Millipore filter. Once in the agar, the non-covalently bound partner molecules separate, and the much less water soluble iodinin takes the form of either oil droplets or crystals. An alternative idea is that there is a water-soluble precursor, chemically distinct from iodinin, which is released by the cells and is able to pass through the cellophane and accumulate in the agar medium. In the medium it undergoes a condensation reaction to form iodinin, either spontaneously and not enzymatically, or by the catalytic action of an exo-enzyme released by the bacterium. The much less water soluble iodinin then separates from the aqueous medium as either an oil or as crystals. For more details, see Appendix C.

##### Identification of the Material as Iodinin

**Figure 7:**
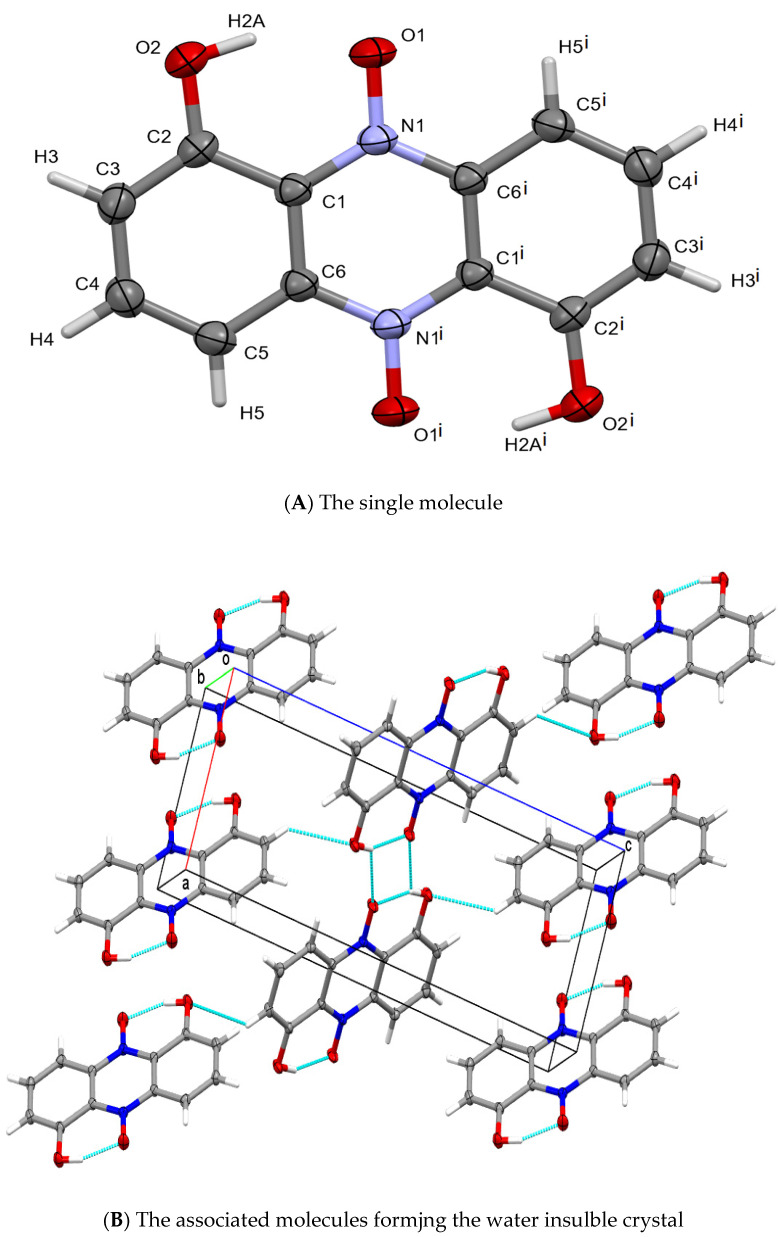
Representations of the chemical and crystallographic structure of iodinin. **Legend:** The chemical structure of the colored component (iodinin) of the exocrystal produced by *X. szentirmaii*. (**A**) the single crystal structure of iodinin. Crystallographic parameters: Monoclinic, P2_1_/c: a = 6.0298(5), b = 5.0752(4), c = 15.854(1) Å, b = 90.421(2)°. Crystal size: 0.48 × 0.15 × 0.02 mm. θ range: 2.57 to 28.28°. Data/restraints/parameters: 1206/0/83. GooF: 1.178. R values [I > 2σ(I)]: R1 = 0.0699, wR2 = 0.1659. (**B**) packing of iodinin in the solid-state is dominated by π-stacked layers connected by C-H⋅⋅⋅O intercations making it largely insolubile in water. The structure of iodinin (from another organism) was previously reported [118].

Crystals were finally isolated using a double layer of sterile cellophane covering an LB plate, and over-layered with a bacterium suspension. Using single crystal X-ray diffraction, the pigment crystal was identified by Haynes and Zeller as iodinin [24], (see also in Figure 7). Details of the structure determination are given in Appendix C and Figure 7. For references see [124,125,126,127,128,129,130,131].

##### Crystal Mutants in *X. szentirmaii* DSM16338T (EMC)

Fodor carried out Tn-mutagenesis experiments, screening for exocrystal-minus mutants. A total of 22 anti-microbial crystal mutants from *X. szentirmaii* were isolated. Some of these can be seen in Figure 20 in the article [86]. One mutant produced colorless oily drops (on the left side), others produced dark oils (in the right of the picture) while the wild type produced purple colored (Medium) oily drops on ENGM plates, (see Appendix B). The mutants were deposited in the stock collection of Professor Heidi Goodrich-Blair (University of Wisconsin, Madison, WI, USA). The Bode Laboratory recently discovered diversity-oriented modifications of the phenazine core through two distinct BGCs in *X. szentirmaii.* A previously unidentified aldehyde intermediate, which can be modified by multiple enzymatic and non-enzymatic reactions, is a common intermediate bridging the pathways encoded by the respective biosynthetic gene clusters BGCs [132].

**The Discovery of a Unique Phenazine Biosynthesis Pathway in *X. szentirmaii* by the Bode Laboratory.**

From an antiSMASH22 analysis of 28 *Xenorhabdus* and *Photorhabdus* genome sequences in the Bode Laboratory, four strains encoding phenazine BGC(s) were identified, but only *X. szentirmaii* from our laboratory encoded two phenazine BGCs [25]. The second BGC was silent under laboratory conditions. The first includes 7 genes (A, B, C, D, E, T, F) with the same transcription orientation (5′–3′) as for the phenazine core biosynthesis. This is followed by gene U, of unknown function, and opposite transcription, followed by gene V, of unknown function, but similar transcription (orientated as A–F), finally followed by genes G and H, encoding for iodinin biosynthesis, [25]. and maintaining the same transcription (5′–3′) orientation as A–F [132]. Although the authors specifically pointed this out, please note that in this pathway, unlike the second, no NRPS-like enzyme-coding gene is represented.

## 4. A Discussion: Discoveries and Evaluation

### 4.1. Strain and Genomic Information

All data and information discussed in this subchapter are given Table 1.

#### 4.1.1. Strain, and Genomic Information on *Xenorhabdus szentirmaii*

**About EPN symbionts**: The only EPB symbiont published so far was *X. szentirmaii* nov. DSM16338T [9]. Nobody has published a paper saying that the natural EPB symbiont of her or his isolate was not *X. szentirmaii* [136,137]. Until recently, we had not found any report of isolation *X. szentirmaii* from an EPN other than *S. rarum*. Last year, however, Castaneda-Alvarez and associates [138], discovered one single, motile, Gram-negative, and non-spore-forming, rod-shaped symbiotic bacterium, strain VLST, isolated from the EPN *S. unicornum* in Chile. Based on the 16S rDNA sequence analysis, the closest related species to the VLST isolate is *X. szentirmaii*. However, deeper analyses, using the whole genome for phylogenetic reconstruction, indicates that VLST exhibits a unique clade in the genus, suggesting a new species, *X. lircayensis* sp. nov. (type strain VLST = CCCT 20.04T = DSM 111583T) [138].

**Genome Information related to *X. szentirmaii*:**

*Xenorhabdus szentirmaii* nov. Type strain, DSM16338(T) (EMC), was identified in 2005 [9]. Draft Genome Sequence and Annotation of this Entomopathogenic Bacterium *X. szentirmaii* Strain was made and published by Gualtieri and his associates in 2014 [99].

The Genome announcement confirms that *X. szentirmaii* is an important producer of antimicrobial activity, as noted by several authors [67,139,140,141].

The genomic DNA was purified [142]. The sequencing strategy was conducted by GATC Biotech (Konstanz, Germany), and a mixed sequencing strategy with Roche 454 GS-FLX titanium and Illumina technologies was followed [139]. The final assembly consisted of 164 contigs, comprising a total length of 4.84 Mb (4.82 Mb without undetermined bases), and has a 43.98% GC. [140]. Functional annotation was carried out using tools of the MicroScope platform [143]. The assembly of *X. szentirmaii* contains 4794 genomic objects, including 4680 coding sequences, 4 rRNA genes, 58 tRNA genes, and 23 noncoding RNAs. Genome annotation highlighted the presence of 71 genes encoding nonribosomal peptide synthetases, and polyketide synthases in *X. szentirmaii*. Therefore, this bacterium is a promising reservoir for non-ribosomal synthesized peptides with new bioactive effects, such as antimicrobial activities.

#### 4.1.2. Strain, and Genomic Information on *Xenorhabdus budapestensis*

EPN symbionts of *Xenorhabdus budapestensis* isolates: wherever and whenever an EPB symbiont of *S. bicornutum* was identified, it was always *X. budapestensis* [11]. We did not find any data to the contrary in the literature. Type strain DSM16342(T) [9] was isolated from *S. bicornutum*, obtained from soil in Central Europe, at the Hungarian–Serbian border [11], and was deposited in Ralf Ehlers’ Lab, Braunschweig, Germany.

The Chinese isolates are from the soil of Inner Mongolia. Strain D43, which was designated HIP57, was found in 2012 [87]. Strain NMC-10 was found in 2012 [133], and strain SN84 was found in 2018 [92,133]. The nematode hosts were not noted. Later, strain C72 was sequenced in 2021 [134], and was from nematodes “belonging to the *S. bicornutum* group”. The sequences of bacterial recA and gyrB genes have shown that the symbiont of *S. pakistanense* is closely related to *X. indica*, which is associated with some other nematodes from the bicornutum group [111], but the authors did not refer to the type strain, DSM16342(T) (EMA). We sequenced neither recA nor gyrB genes from DSM16342(T), while Bath and associates [111], have not sequenced the 16SrDNA of either of their bacteria, so the available molecular information does not allow us to conclude regarding any similarities or differences.

**Genome Information Related to *X. budapestensis*:**

The genome sequence of *X. budapestensis* Nov. Type Strain DSM16342(T) has been available in the XenorhabduScope database, https://www.genoscope.cns.fr/agc/microscope/home/index.php, (accessed on 25 February 2022). It was deposited by Prof. Helge Bode (personal communication). The announcement of the high-quality, complete, and annotated genome sequence of *X. budapestensis* strain C72 reports 15 secondary metabolite biosynthetic gene clusters identified in the genome. These are responsible for the production of a diverse group of antimicrobial compounds to help host plants against agricultural pathogenic diseases [134].

### 4.2. AMP Products of X. budapestensis and X. szentirmaii

All data and information discussed in this subchapter are presented in Table 2.

These EPB species are a promising reservoir for non-ribosomal synthesized peptides with new bioactive effects, such as antimicrobial activities. The antimicrobial-active *Xenorhabdus* (PAX)-peptides discovered in and redundantly produced by EMA and EMC, and discussed above, are promising candidates for controlling MDR pathogens (including bacteria, oomycetes, fungi, and protozoa) [144].

#### 4.2.1. A List of AMPs from *Xenorhabdus* Species Other Than *X. budapestensis* and *X*. *szentirmaii*

The list includes the above-mentioned lysine-rich cyclo-dipeptide family from *X. nematophila* [99,103]. Lys-rich PAX lipopeptides are also produced by *X. khoisanae* SB10 [144,149].

Also included are the antifungal cabanillasin, produced by *X. cabanillasii* JM26 [150], taxlllaids (A-G) produced by *X. indica* [151], xenortids from *X. nematophila* [152,153], and xenocoumacins from *X. nematophila* [154].

A major issue currently facing medicine is antibiotic resistance. No new class of antibiotics for the treatment of Gram-negative infections has been introduced for some time [155].

A competitive French research team screened a collection of *Xenorhabdus* and *Photorhabdus* EPB strains in the quest to discover new structures that are active against the most problematic multidrug-resistant bacteria. Odilorhabdins (ODLs), a novel antibacterial class, were identified from this research. These compounds inhibit bacterial translation by binding to the small ribosomal subunit at a site not exploited by current antibiotics [156].

Based on structure-activity relationship, and studies on the inhibition of the bacterial translation of novel Odilorhabdins analogs, the problem of developing the total synthesis of this family of peptides was resolved. A medicinal chemistry program was started to optimize their pharmacological properties. NOSO-502, the first ODL preclinical candidate, was selected [157]. This compound is currently under preclinical development for the treatment of multidrug-resistant Gram-negative infections in hospitalized patients [155]. The recently published review by the South African *Xenorhabdus* team is highly recommended to readers [158].

#### 4.2.2. Antimicrobial Products of *Xenorhabdus szentirmaii* Other Than the Fabclavines and Phenazines

**Xenofuranone A and B** (phenylpyruvate dimers) were the first AMPs identified from *X. szentirmaii* [146]. Xenofuranones have been isolated from the CFCM of *X. szentirmaii*, and their structures were elucidated by NMR and mass spectroscopy. Both compounds resemble fungal furanones, and their biosynthesis was elucidated using a reversed approach. Putative ^12^C precursors were fed to an overall ^13^C background in small-scale experiments, followed by gas chromatographic analysis coupled to mass spectrometry [146].

**Szentiamide,** as a new cyclic hexadepsipeptide, was isolated from the CFCM of *X. szentirmaii* [147,148]. The structure was revealed by analysis of one- and two-dimensional NMR spectra, and high-resolution mass spectrometry. The amino acids were determined to be D-leucine, L-threonine, D-phenyl-alanine, D-valine, L-tyrosine, and L-tryptophane, after hydrolysis and derivatization with D-FDVA [Nalpha-(2,4-dinitro-5-fluoro-phenyl)-D-valinamide] from *X. szentirmaii* [147]. The total chemical synthesis of the depsipeptide szentiamide has been completed [148]. The compound derived from the efficient synthesis enabled additional bioactivity tests leading to the identification of a notable activity against insect cells and plasmodium [148].

**Rhabdopeptide**/**Xenortide-like Peptides** were confirmed by a recent publication from the Bode Laboratory [159].

#### 4.2.3. Antimicrobial Products of Strains of *X. budapestensis* Other Than DSM 16342T (EMA)

The list of antimicrobial peptides from strains other than the types strain EMA of *X. budapestensis* includes AMP molecules GP-19 and EP-20, active against plant pathogenic *Verticillium dahliae* and *Phytophthora capasicae*, respectively, and produced by *X. budapestensis* NMC-10 [133], and xenematides F and G (depsipeptides) from *X, budapestensis* SN84 [134]. Rhabdopeptides were also isolated from SN84 [92].

## 5. Conclusions

This review deals with the scientific impact and perspectives provided by EPB symbionts in EPN/EPB symbiotic associations producing beneficial antimicrobial compounds, and focuses special attention on two entomopathogenic bacterium species, *X. budapestensis* (EMA) and *X. szentirmaii* (EMC), which are natural obligate symbionts of EPN species *S. bicornutum* and *S. rarum*, respectively. They have been discovered, described and characterized in our laboratories (Department of Genetics, Eötvös University in Budapest, Hungary; and DSMZ Braunschweig, Germany, headed by Erko Stackebrandt) [9].

Our unofficial “International Laboratory without Walls” where we worked on EPN/EPB Research also included facilities in Milwaukee, WI, USA, (those of Kenneth H. Nealson, Steven A. Forst), in Wooster, OH (Michael G. Klein), at USDA, Beltsville (David Chitwood), and the Kossuth University, Debrecen, Hungary. From there, the late Professor Attila Szentirmai coordinated the international cooperative research, within the frame of the European COST 819 and COST 850 Actions, and the US-Hungarian Joint Fund. In tribute to this great personality, we decided to write this review. The draft sequence of EMC was determined and annotated in Nimes, France by Maxime Gualtieri, which let the project survive and is not be forgotten. Each designated strain of *X. budapestensis* (DSM16342)(T), C72, SN84, NMC110), the only designated strain of *X. szentirmaii* (DSM16338)(T), along with the undesignated strains, proved to be excellent antibiotic producers. The question is—what does this mean from an evolutionary point of view?

Evolutionary experiments indicate that selection for maintenance of mutualism has always been stronger than selection for increased virulence of the EPB [71]. The contribution to the cospeciation of the EPN partner is also important. IJ larvae of *Steinernema* species harbor their EPB symbionts in a discrete structure located in the anterior portion of their intestine known as the ‘bacterial receptacle’ (formerly known as the bacterial or intestinal vesicle). At the morphological level, species can be grouped into two categories based on the presence or absence of vesicles within the receptacle [160]. Our experience is that the symbiotic EPB-partner exchange between taxa members of the *S.’feltiae’* (clade III, characterized by having a vesicle) usually does not cause any problem [161]. The *Steinernema-Xenorhabdus*–insect partnerships are extremely diverse and represent a model system in ecology and evolution with which to investigate symbioses between invertebrates and microbes. The reproductive fitness of the nematode-bacterium partnership is tightly associated, and maintenance of their virulence is critical [162].

On the basis of the available data on *S. bicornutum* [11], *X. budapestensis* and *S. rarum* [10], and *X. szentirmaii*, symbioses seem to be fixed co-evolutionarily in their respective niches. Their competitiveness is definitely based on their powerful AMP arsenal, not the R-type bacteriocins [97]. However, it cannot be excluded that in other niches, or in other geographic locations, the respective EPN species could or could not establish stable symbioses with other *Xenorhabdus strains.*

The sequences of the recA and gyrB genes have shown that the symbiont of *S. pakistanense* is closely related to *X. indica*, which is associated with some other nematodes from the bicornutum group [111], but the authors did not refer to the type strain EMA [9]. Since we did not sequence either recA or gyrB genes of EMA, and Bhat and associates also did not sequence the 16SrDNA of their EPB, no conclusions about their relatedness can be made.

*Steinernema costaricense* (Panagrolaimorpha: Steinernematidae) was discovered from the Bush Augusta State Park, MOI, USA [163]. Morphologically it seemed very similar to *S. costaricense* from Costa Rica. Based also on high similarity of their bacterial symbionts, the new isolate was identified as *S. costaricense*, and suggested phylogenetic affinities between *S. costaricense* and the bicornutum group [163]. Later however, by using all three available methods of analysis for the EPN phylogenetic marker ITS region, sequences showed that four species *of Steinernema* from the Americas *(S. rarum* [10]; *S. scarabaei* [162] *S. unicornum* and *S. costaricense* Missouri isolate) formed only a weakly supported clade [163]. The Missouri isolate never formed a clade with either *S. rarum* or *S. unicornum* [164]. Neither of the two publications states exactly what “high similarity of their bacterial symbionts” means.

What we know for certain is that the natural symbiont (DSM16342(T)) of *S. bicornutum* [11], and DSM16338T of *S. rarum*, [10] cannot replace each other as symbionts [161], and on the basis of 16SrDNA information, they do not form a “clade” [9].

Those references which appeaed during refreshing the manuscipt are commented in Appendix D.

## 6. Closing Remark

In Memoriam Professor Attila Szentirmai.

**Figure 8:**
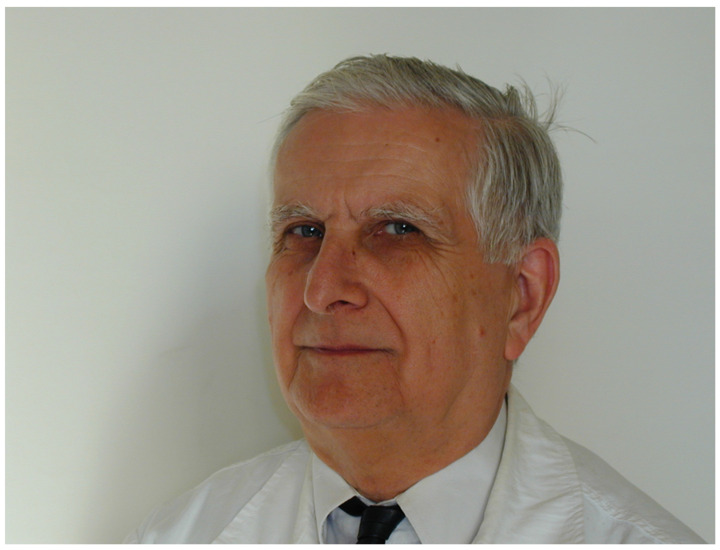
Professor Attila Szentirmai. (1930–2019).

An unconventional goal in this review is to serve as a requiem to Professor Attila Szentirmai (Figure 8), whose accomplished, fruitful, and active life came to an end in 2019. He was the father of EPN/EPB research in Hungary, and was an outstanding scientific expert in industrial microbial biotechnology.

He has been internationally acknowledged as a pioneer of antifungal antibiotic research, as these publication milestones indicate [165,166,167,168,169,170,171,172,173,174,175].

He was the founder of industrial microbial biotechnology in Hungary, the homeland of internationally known and acknowledged pharmaceutical companies (Gedeon Richter; Chinoin; Biogal). Biochemical engineers had been educated only in the Technical University in Budapest before then. However, thanks to the appointment of Professor Szentirmai as Head of the Microbiology and Biotechnology Department of the (Kossuth) University in Debrecen in 1985, in the framework of MSc and PhD programs, well-trained biologists were educated and employed by the industry as respected biotechnological engineers.

Attila was the author, or co-author, of over 60 scientific publications, and more than 40 patents used in the pharmaceutical industry. As for research orientation, his main profile has always been the pharmaceutical industry, but he was the catalyst for introducing, establishing, and continuing EPN/EPB research in Hungary.

As for his relation to EPN/EPB research, it was initiated by K.H. Nealson and S.A. Forst, via A. Fodor. In the beginning, this research trend in Hungary was tolerated rather than favored, but later took off and grew through the professional guidance and support of Professor Szentirmai. With his help, it has become an esteemed research project in the country. He is a co-author of several papers in the field [24,59,60].

As for his personality, if one tried to compare him to someone from the Bible, this person must be “Job”; and if one tried to compare him to an internationally known scientist, this person must be John Sulston. The difference between the two was that Attila, the “Hungarian John Sulston”, was incurably practice-oriented, so a Nobel Pprize for him was always out of the question. The English John Sulston was an atheist, while Attila was a believer, but their mentalities towards other people were identical. However, their main common feature was the capability “to win without fighting, without even competing”, but just by doing excellent research.

He claimed that he believed in ever-lasting life in the memory of people. Professor Szentirmai was very sorely tested several times in his life, but he always managed to remain steadfast and strong at his home, at the bench, and in the classroom. He was a great teacher. He survived and remained productive in a country where, at that time, political capital provided an enormous advantage to those who had it without having it. He was always indispensable and reliable. What he discovered worked in industrial practice.

Fellow scientists were frequently amazed at his consistency and research acumen: his resources of the previous grants, beginning the new project with preliminary experiments, and tested hypotheses. His patented inventions were not made for his drawer, but for the most successful pharmaceutical factories in his country. The antibiotics he discovered have been widely used as drugs.

The scientific output of the younger generations working in his previous Department is also impressive (e.g., [169,170]. One of the co-authors (L. Karaffa), is the successor of Professor Szentirmai, and continues the work of his predecessor.

## Figures and Tables

**Table 1:** An Inventory of Deposited Strains of *Xenorhabdus szentirmaii* and *X budapestensis* available for research. *Xenorhabdus budapestensis* Type strain, DSM16342(T) (EMA), was isolated from the Central European isolate of *Steinernema bicornutum* [11], and identified in [9]. The sequence was determined, and the sequence information was deposited by Prof. Helge Bode (personal communication, available at https://www.genoscope.cns.fr/agc/microscope/home/index.php, (accessed on 25 February 2022) but not published). *Xenorhabdus budapestensis* Strain D43, which was designated HIP57, was found in 2012 [87]. *Xenorhabdus budapestensis* Strain NMC-10 was identified in 2012 [133]. *Xenorhabdus budapestensis* Strain SN84 was identified in 2018 [92]. This was the source of rhabdopeptide and depsipeptides (xenematide F and xenematide G) [134]. Lately, strain C72 was isolated from nematodes “belonging to the *S. bicornutum* group”, and was sequenced [135]. The complete genome sequence for C72 has been deposited into GenBank under accession number CP072455 (genome annotation is available at https://www.ncbi.nlm.nih.gov/nuccore/2021543890/), (accessed on 25 February 2022), *Xenorhabdus szentirmaii* Type strain, DSM16338(T) (EMC), was identified in 2005 [9]. The Draft Genome Sequence and Annotation of this *X. szentirmaii* Strain was made by [99], doi: 10.1128/genomeA.00190-14. *PMID: 24625876; PMCID: PMC3953197.* The annotated genomes were implemented in the public XenorhabdusScope database https://wwwgenoscope.cns.fr/agc/microscope/home/index.php, (accessed on 25 February 2022).

Bacterium EPB Species	Strain		Nematode		Genome Information	
		Ref	EPN Partner	Ref	Genome Announcement	Ref
*Xenorhabdus budapestensis*	DSM16342(T)	[9]	*S. bicornutum* Central Europe, 1995	[11]	Bode H (https://www.genoscope.cns.fr/agc/microscope/home/index.php (accessed on 25 February 2022)).	-
	D43, designated as HIP57	[87]	*S. bicornutum* China, 2012	[87]	-	-
	NMC-10	[133]	*S. bicornutum* China, 2012	[133]	-	-
	SN84	[92]	*S. bicornutum* China, 2018	[92]	-	-
	SN84	[134]		[134]	-	-
	C72	[135]	*S. bicornutum* China, 2021	[135]	https://www.ncbi.nlm.nih.gov/nuccore/2021543890/ (accessed on 25 February 2022).	[99]
*Xenorhabdus szentirmaii*	DSM16338(T)	[7]	*S. rarum*, Cordoba, Argentina, South America	[8]	implemented in the public XenorhabduScope database (https://wwwgenoscope.cns.fr/agc/microscope/home/index.php (accessed on 25 February 2022)).	[116]

Abbreviations: X = *Xenorhabdus*; *S*. = *Steinernema*; Ref = references.

**Table 2:** An Inventory of Antimicrobial Peptides (and Related References) Produced by *Xenorhabdus szentirmaii* and *X. budapestensis* and Discussed in This Review. Both *Xenorhabdus budapestensis* (DSM16342)T (EMA) and *X. szentirmaii* (DSM16338)T (EMC) [9], produce strong PAX peptides, including arginine-rich peptides of a short carbon chain, with or without detectable antimicrobial activities [18]. The most active antimicrobial among them is fabclavine and its derivatives [19]. The draft genome sequencing of EMC [139] demonstrated that the assembly of *X. szentirmaii* EMC contains 4794 genomic objects, including 4680 coding sequences, 4 rRNA genes, 58 tRNA genes, and 23 non-coding RNA genes. Genome annotation highlighted the presence of 71 genes encoding nonribosomal peptide synthetases and polyketide synthases in *X. szentirmaii* DSM16338. This indicates a promising reservoir for non-ribosomal synthesized (NRS) peptides with new bioactive effects. *Xenorhabdus* *szentirmaii* EMC produces xenofuranones A and B [145,146,147], iodinin [24,86,132], szentiamide [148,149], and phenazines [132]. *Xenorhabdus budapestensis* NMC-10 produces two novel antimicrobial peptides with antibacterial and actinomycete activities [133]. *Xenorhabdus budapestensis* SN84 produces two cyclic depsipeptides called xenematides F and G [134], and rhabdopeptides with nematicidal activities against plant pathogenic nematode *Meloidogyne incognita* [92]. The high-quality, complete, and annotated genome sequence of *X. budapestensis* strain C72 revealed 15 secondary metabolite biosynthetic gene clusters identified in the genome that are responsible for the production of a diverse group of antimicrobial compounds [135]. The pioneering work leading to the discovery of PAX peptide is described in [18].

*Xenorhabdus*		Biosynthetic Operons, Antimicrobial Products	Reference
Species	Strain		
*szentirmaii*	[9] Lengyel et al., 2005 DSM16338T (EMC)	Draft Genome Annotation,	[139]
		Iodinin	[24,86,132];
		Phenazine	[132]
		Xenocoumacines	[145,146]
		Szentiamide,	[147,148]
		Fabclavine	[9,18,19,20,21,22]
*budapestensis*	[9] Lengyel et al., 2005 DSM16342T (EMA)	Fabclavine, Bicornutin	[7,18,19]
	[135] Li et al., 2021 C72	Genome Annotation	[135]
	[133] (Xiao et al., 2012) NMC10	GP-19, EP-20	[133]
	[134] Xi et al., 2021 SN84	xenematides F xenematides G(depsipeptides	[134]
	[92] Bi et al., 2018	Rhabdopeptides	[92]
	[87] Yang et al., 2012	Insecticidal protein	[87]

## Appendix A

Reliable and Reproducible technique for Isolation EPB Primary Cells from Surface- Sterilized EPN IJ. This is the Legend for Figure 2.

Isolation of entomopathogenic bacteria cells from the monoxenically colonized gut of surface-sterilized, EPN, infective dauer juveniles (IJ) of their symbiotic partner by the bleach method of Attila Lucskai, was first published in [9]. The Lucskai technique was used by us for many years after being modified as follows. Under a dissecting stereomicroscope, 10–15 IJ were transferred with a platinum wire into 1% HOCl, forming a drop on the inner surface of the lid of a sterile Falcon Petri plate, and incubated for 30–60 days. IJs were then transferred one by one, into a consecutive series of 100 μL of M9 solution (Minimal Salts, Sigma-Aldrich, Budapest, Hungary) [6] with a flamed-and-cooled platinum wire to remove the excess of the HOCl. IJs were then cut with the sterilized platinum wire in the last drop of M9, which were then transferred and spread on the surface of LB agar. The plates were incubated at 30 °C. Usually, up to 15 colonies developed within 1–2 days. The bacterial colonies were transferred onto indicator plates (nutrient bromothymol blue agar (NBTA) or Luria Broth) containing 1 mL of 25 μg/mL Bromothymol Blue (Sigma-Aldrich) and 1 mL of 40 μg/mL 2,3,5-triphenyltetrazolium chloride (Sigma-Aldrich) for another 2 days at 28–30 °C. Only the blue colonies were considered to be the symbiont. Usually, no other bacteria grew on the plates. At least eight replicas were made, one of which was subsequently used in investigations. Each strain mentioned in this review was isolated in our lab in Budapest, and the EPB identifications from the gut of surface-sterilized EPN IJ, were established based on 16S rDNA sequence at the Laboratory of DSMZ in Braunschweig run then by Professor Stackebrandt.

## Appendix B

The ENGM Media for Reproducible Gnotobiological Studies.

This is a Legend to Figure A1: ENGM (Entomopathogenic Nematode Growth Media): an NGM-like solid media suitable for doing genetics on the entomopathogenic nematodes. The recipe of ENGM is as follows: 2.5 g bacto-peptone; 1.5 g beef extract; 2.3 g brain-heart infusion; 15 g agar to 1 L of deionized water. After autoclaving: 5 g vegetable oil; 1 mL of 5 mg/mL cholesterol (dissolved in EtOH); 2 mL of 0.5M MgSO4;. When needed, antibiotics (rifampicin 100; dissolved in alkalized methanol; and kanamycin 30, sterile filtered, were added after cooling the autoclaved media before solidification). ENGM plates could be seeded with moderately growing symbiotic bacteria, such as NS107. (For details on the isolation of NS107, please contact the corresponding author). We elaborated the ENGM so that both EPN species and *Cenorhabdus elegans*, as well as their food-source bacteria (*Photorhabdus luminescens* TT01, *Xenorhabdus szentirmaii* DSM16338(T) *Escherichia coli* OP50) could properly grow. The visibility of the nematodes on ENGM is almost as good as that on NGM [86].

## Appendix C

Chemical Structure Information on Iodinin.

This is important supplementary information to the sub-section (Identification of the material as iodinin by Matthias Zeller), and additional new information related to research efforts on solubilization of the anticancer iodinin to improve its bioavailability.

Diffraction data were collected on a Bruker Smart APEX diffractometer at 298 K using monochromatic Mo Kα radiation with the omega scan technique. Data for the sample were collected and its unit cell was determined using SMART 5.630 [1]; the data were integrated using SAINT V8.40B [2] and corrected for absorption and other systematic errors using SADABS 2016/2 [3]. The space group was assigned using XPREP [4]; the structure was solved by direct methods using ShelXS-97 [5], and refined by full-matrix least-squares against F^2^ with all reflections using Shelxl 2018-3 [6] and ShelXle [7]. Hydrogen atoms attached to carbon were positioned geometrically, and constrained to ride on their parent atoms, with carbon-hydrogen bond distances of 0.93 Å. Positions of hydroxyl H atoms were freely refined. Uiso(H) values were set to a multiple of Ueq(C/O) with 1.5 for OH and 1.2 for C-H units, respectively. Complete crystallographic data, in CIF format, have been deposited with the Cambridge Crystallographic Data Centre. CCDC 2150298 contains the supplementary crystallographic data for this paper. These data can be obtained free of charge from The Cambridge Crystallographic Data Centre via www.ccdc.cam.ac.uk/data_request/cif (Assessed on 15 Feburary 2022) For references, see [124,125,126,127,128,129,130,131]. The X-ray diffractometer was funded by NSF Grant CHE 0087210, Ohio Board of Regents Grant CAP-491, and by Youngstown State University.

The crystal structure of iodinin had previously been described [118], and the crystallographic aspects of the structure of iodinin had been discussed in detail in the original report. In the article by Hanson and Hum [118], the authors point out the presence of impurities that had been partially deoxygenated at the N-oxide positions, in the original sample of biosynthetic origin, and a second purer sample needed to be obtained for structure determination. Interestingly, no such difficulties have been observed when conducting the present iodinin structure determination, pointing towards the purity and efficiency in which iodinin is produced by *X. szentirmaii* EMC [9]. The biological significance of iodinin has recently been rediscovered [165,166,167] and its near insolubility in aqueous solutions needs to be overcome [123]. Phenazine is known to regroup planar nitrogen-containing heterocyclic compounds, and can be used to enhance the bioavailability of iodinin. Its water solubility has led to the development of new formulations using diverse amphiphilic α-cyclodextrins (CDs). Per-[6-desoxy-6-(3-perfluorohexylpropanethio)-2,3-di-O-methyl]-α-CD were recently successfully used to obtain iodinin-loaded nano-formulations with good parameters [123]. We believe that EMC has a natural recipe of iodinin solubilization.

**Supplementary material to original observations on iodinin crystal phenotypes:** Here we refer to [86], and the Figures S18–S20, published there. In Figure S18 EMC colonies on LBA (Left) and LBTA (Medium) plates are shown. A 40 × magnification of the crystals on the surface of a colony, as seen through Leica stereo-microscope, is depicted on the right pane of Figure S18. In Figure S19., one can see a light microscopy image, at 125 × of an isolated antibiotic condensed, water-insoluble iodinin crystals under the agar (D), and an electron microscopy image at 1000 × (E) (SEM, S-4700 20.0 kV 11.1 mm × 4.99 SE. (Photo: A. Máthé-Fodor).

In repeated experiments, when EMC was grown on a Millipore cellulose filter of 0.22 µm pore size, the crystals also appeared under the filter, and a diffusion zone of a strong purple color appeared on the filter. The material was extremely hydrophobic and insoluble in ethanol. Using acetonitrile or chloroform, a colored compound having an absorption maximum in the UV range can be separated from a colorless, insoluble material. Dianne K. Newman and Heidi Goodrich-Blair suggested that the compound must be a phenazine. This was of particular interest, since they had never seen a purple one before (Heidi Goodrich-Blair, personal communication), prompting further analysis of isolated crystals by single crystal X-ray diffraction.

## Appendix D

Five Historical References from the Literature Representing Milestone Research, not cited in the text. See References for complete information.

References [176,177,178] are the pioneer work leading to the birth of polyphasic taxonomy, based on comparative reproducible analysis of DNA sequences of genes considered to be of no selective value, like 16SrDNA genes of bacteria. This pioneering work came from the Stackebrandt School, Braunschweig, Germany, stimulating our research efforts into gnotobiological studies and NRP-AMPs in our world-wide Laboratory With-out Walls.

Ref. [179] described an excellent way of determination of the absolute configuration of peptide natural products by using stable isotope labeling and mass spectrometry which revolutionized the chemistry of NRP-AMPS produced by EPB species.

Ref. [72], The dauerlarva, was added following the advice of our respected reviewers, as important information for readers who are not nematode specialists.

Refs. [100,180] provide the most recent information about the discovery of the coordinated post-transscriptional regulation of the biosynthesis of chemically completely different “secondary” metabolites of importance for EPN/EPB symbiotic associations.
